# ^15^N-DNA stable isotope probing reveals niche differentiation of ammonia oxidizers in paddy soils

**DOI:** 10.1007/s00253-024-13170-x

**Published:** 2024-05-24

**Authors:** Fuyun Gao, Yaying Li, Haoxin Fan, Dan Luo, Stephen J. Chapman, Huaiying Yao

**Affiliations:** 1 Key Laboratory of Urban Environment and Health, Institute of Urban Environment, Chinese Academy of Sciences, Xiamen, 361021 People’s Republic of China; 2 University of Chinese Academy of Sciences, Beijing, 100049 People’s Republic of China; 3 Research Center for Environmental Ecology and Engineering, School of Environmental Ecology and Biological Engineering, Wuhan Institute of Technology, Wuhan, 430073 People’s Republic of China; 4 College of Petrochemical Engineering, Lanzhou University of Technology, Lanzhou, 730050 People’s Republic of China; 5 The James Hutton Institute, Craigiebuckler, Aberdeen, AB15 8QH UK

**Keywords:** Ammonia-oxidizing archaea, Ammonia-oxidizing bacteria, Comammox *Nitrospira*, ^15^N isotope, Nitrification inhibitor

## Abstract

**Abstract:**

Chemoautotrophic canonical ammonia oxidizers (ammonia-oxidizing archaea (AOA) and ammonia-oxidizing bacteria (AOB)) and complete ammonia oxidizers (comammox *Nitrospira*) are accountable for ammonia oxidation, which is a fundamental process of nitrification in terrestrial ecosystems. However, the relationship between autotrophic nitrification and the active nitrifying populations during ^15^N-urea incubation has not been totally clarified. The ^15^N-labeled DNA stable isotope probing (DNA-SIP) technique was utilized in order to study the response from the soil nitrification process and the active nitrifying populations, in both acidic and neutral paddy soils, to the application of urea. The presence of C_2_H_2_ almost completely inhibited NO_3_^−^-N production, indicating that autotrophic ammonia oxidation was dominant in both paddy soils. ^15^N-DNA-SIP technology could effectively distinguish active nitrifying populations in both soils. The active ammonia oxidation groups in both soils were significantly different, AOA (NS (*Nitrososphaerales*)-Alpha, NS-Gamma, NS-Beta, NS-Delta, NS-Zeta and NT (*Ca.* Nitrosotaleales)-Alpha), and AOB (*Nitrosospira*) were functionally active in the acidic paddy soil, whereas comammox *Nitrospira* clade A and *Nitrosospira* AOB were functionally active in the neutral paddy soil. This study highlights the effective discriminative effect of ^15^N-DNA-SIP and niche differentiation of nitrifying populations in these paddy soils.

**Key points:**

*•* ^*15*^ *N-DNA-SIP technology could effectively distinguish active ammonia oxidizers.*

*• Comammox Nitrospira clade A plays a lesser role than canonical ammonia oxidizers.*

*• The active groups in the acidic and neutral paddy soils were significantly different.*

**Graphical Abstract:**

**Figure:**
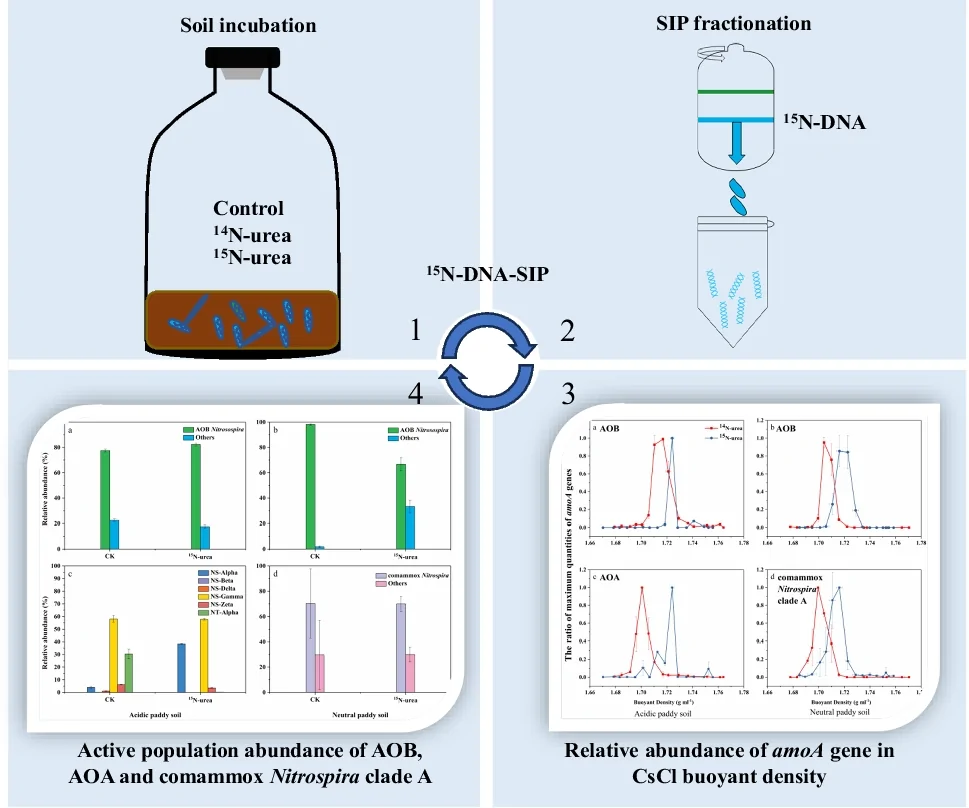

**Supplementary Information:**

The online version contains supplementary material available at 10.1007/s00253-024-13170-x.

## Introduction

Chemoautotrophic nitrifying microorganisms are key players in the global nitrogen cycle (Wang et al. 2020; Sun et al. 2022), and they were traditionally thought to involve ammonia-oxidizing bacteria (AOB) and ammonia-oxidizing archaea (AOA), together with nitrite-oxidizing bacteria (NOB) (Li et al. 2019; Lin et al. 2022; He et al. 2022). With the development of research, the finding of complete ammonia oxidizers (comammox *Nitrospira*), which can catalyze ammonia to nitrate in an independent microorganism, has demonstrated how they can be participants with autotrophic ammonia-oxidizers (Wang et al. 2019a; Li et al. 2019). Up to now, all given comammox *Nitrospira* belong to the sub-lineage II of the *Nitrospira* (Daims et al. 2015; He et al. 2022). Comammox *Nitrospira* can in turn be categorized into two divergent clades, namely clades A and B (Daims et al. 2015; van Kessel et al. 2015), while clade A is further classified into clades A.1, A.2.1, A.2.2, and A.3 (Li et al. 2021a; Sun et al. 2021b; He et al. 2022).

AOA and AOB are widely distributed in all kinds of habitats, which include soils (Ali et al. 2013; Tao et al. 2017), oceans (Wang et al. 2017; Qin et al. 2020), and freshwater (Park et al. 2022; Ren and Wang 2022) and play important roles in global nitrogen and carbon cycles. AOA contain four basal lineages, *Ca.* Nitrosocaldales (NC), *Nitrososphaerales* (NS), *Ca.* Nitrosotaleales (NT), and *Nitrosopumilales* (NP) (Alves et al. 2018). In soil, the activity of AOA and AOB is often linked to pH (Hu et al. 2014; Tago et al. 2015; Ying et al. 2017), perhaps because AOA are oligotrophic microorganisms that prefer acidic conditions (Wu et al. 2017; He et al. 2021), whereas AOB preferred soils with pH higher than 7 (Shen et al. 2012). The ^13^C-DNA-SIP technique is often adopted to study the activity of autotrophic ammonia oxidation (Pratscher et al. 2011; Xia et al. 2011; Pan et al. 2018). AOA, AOB, and comammox *Nitrospira* (nitrifying populations) exhibit differentiated activities (Wang et al. 2019a; Liu et al. 2021). Nevertheless, the technical limitation of ^13^C-DNA-SIP is to determine the autotrophic carbon-fixing growth of nitrifying populations by assimilation of ^13^C (Wu et al. 2013; Pornkulwat et al. 2018; Zhao et al. 2020). Hence, the ^13^C-DNA-SIP technique cannot directly determine the incorporation of N into the genome, although the changes in ammonia and nitrate concentrations are measured in order to characterize ammonia oxidation activity in such experiments (Zhang et al. 2010, 2018; Dong et al. 2019). An alternative technique is DNA-SIP based on ^15^N-substrates, which has employed isotopically labeled N incorporation into the genome to more directly determine active autotrophic microorganisms (Angel et al. 2018; Zhang et al. 2021).

The versatility of metabolism allows microorganisms to adapt to changes in substrate content or pH (Daims and Wagner 2018). Moreover, recent studies have shown that comammox *Nitrospira* responds positively to an increase in nitrogen substrates (Li et al. 2019, 2020; Osburn and Barrett 2020; Takahashi et al. 2020). Likewise, not all AOA were limited by high ammonia concentrations, and some AOA like *Nitrosocosmicus* AOA are similar to AOB and have been demonstrated to tolerate very high ammonium concentrations (Jung et al. 2021). In addition, kinetic analysis has shown that terrestrial AOA and AOB have similar ammonia affinities (Bello et al. 2021), which may be the cause for the vitality of AOB in acidic soils. More and more evidence has shown that nitrification in acidic soils is chiefly carried out by AOA (Gubry-Rangin et al. 2010; Zhang et al. 2012; Lu et al. 2012; Lu and Jia 2013). However, there are also AOB in acidic soils and the mechanism of their growth has been illustrated in low pH soils (Zhang et al. 2017; Huang et al. 2018; Séneca et al. 2020; Picone et al. 2021). In the presence of nitrification inhibitors (NIs) like C_2_H_2_, reducing competition for ammonia may also enhance any selective advantage provided by high ammonium (Ye et al. 2018).

The purpose of this experimentation was to investigate the relative contribution of nitrifying populations to nitrification, as well as their ecological niches in fertilized paddy soils. We set up a laboratory microcosm experiment to (1) measure the responses of nitrifying populations to the addition of nitrogen fertilizer and the nitrification inhibitor (C_2_H_2_) in both acidic and neutral paddy soils, (2) estimate the incorporation of ^15^N into the genome DNA of nitrifying population, and (3) analyze the active communities of ^15^N-incorporating nitrifiers.

## Materials and methods

### Soil sites

The acidic paddy soil (pH_H2O_ 5.19) was collected in Yingtan (116° 55′ E, 28° 12′ N), Jiangxi Province, China. The mean annual precipitation (MAP) of the acidic paddy field is 1785 mm, and the mean average temperature (MAT) is 17.8 °C. The neutral paddy soil (pH_H2O_ 7.09) was sampled in Baoshan (99° 15′ E, 25° 09′ N), Yunnan Province, China. The MAP of the neutral paddy field is 1000 mm, and the MAT is 16.0 °C. The soil samples were sampled from 0 to 20 cm using eight random soil cores (diam. 10 cm) and mixed into composite samples. Then, they were transported to the laboratory on ice for subsequent research and analysis. The determination methods for basic soil physicochemical properties were as described in previous studies (Li et al. 2021a, b; Yu et al. 2022; Zhang et al. 2022) and given in Table 1.

**Table 1:** Basic physicochemical properties of acidic and neutral paddy soils

ID	pH	TC (g/kg)	TN (g/kg)	C/N	NH_4_^+^-N (mg/kg)	NO_3_^−^-N (mg/kg)	AP (mg/kg)	AK (mg/kg)	DOC (mg/kg)	Soil texture (%)		
										Clay	Silt	Sand
Acidic paddy soil	5.19	1.89	0.16	12.05	19.78	1.95	43.97	191.18	68.56	19.00	59.00	22.00
Neutral paddy soil	7.09	5.50	0.30	18.31	4.59	20.05	21.61	357.64	105.62	1.25	11.39	87.36

*TC* total carbon, *TN* total nitrogen, *C/N* ratio of carbon and nitrogen, *AP* available phosphorus, *AK* available potassium, *DOC* dissolved organic carbon

### Soil incubation

Four treatments were set up: (1) control without adding any substrate, called CK; (2) ^14^N-urea treatment to provide key confirmation of active organisms in the ^15^N-treated samples; (3) ^15^N-urea treatment with ^15^N-labeled urea (99 atom%; Cambridge Isotope Laboratories, Inc., Andover, MA, USA); and (4) ^15^N-labeled urea and C_2_H_2_ treatment with ^15^N-labeled urea and 0.1% (v/v) C_2_H_2_. In each microcosm incubation system, 20 g of dry-weight soil (DWS) was placed in brown serum bottles (120 mL), equipped with rubber stoppers and aluminum caps for easy sealing. To avoid ammonia loss brought by the rapid hydrolysis of urea, urea was added four times, with each addition being 100 mg kg^−1^ N. Each treatment was sampled at 0 and 28 days, with three replicates per treatment. Aerobic conditions were maintained by opening the rubber plugs and replacing fresh air every 3 days. Incubation was continued for 28 days in a dark incubator at 25 °C. On the day of sampling, the soil was taken to detect the ammonium (NH_4_^+^-N) and nitrate (NO_3_^−^-N) contents. The soil NH_4_^+^-N and NO_3_^−^-N contents were determined by a continuous flow injection analyzer (FLA star 5000 Analyzer, Foss, Denmark) extracted by 1 mol L^−1^ KCl (soil/KCl, 1:10).

### Nucleic acid extraction and SIP fractionation

After freeze-drying, about 0.5 g of soil was taken to extract genomic DNA using the Fast DNA SPIN Kit for Soil (MP Biomedicals, Santa Ana, CA, USA), then immediately aliquoted and placed at − 20 °C for subsequent experimentations.

In order to follow the smooth progress of the ^15^N-DNA-SIP experiment, the initial values of soil ^15^N (acidic paddy soil: 18.92, neutral paddy soil 9.08) were measured by isotope ratio mass spectrometer (Thermo Fisher Scientific, USA). The steps of DNA-SIP were implemented using a previously described method with improvements (Luo et al. 2021). Simply put, 3 µg DNA was added to the liquid mixture which was mixed with CsCl solution and gradient buffer. The liquid mixture was regulated to a refractive index of 1.4015 and centrifuged at 182,742 × g (45,000 rpm) for 48 h at 20 °C (Luo et al. 2021). After centrifugation, 16 × 330 µL was collected into 1.5 mL centrifuge tubes. Next, 100 µL liquor in each centrifuge tube was measured, in order to determine the buoyant densities.

The purification steps of each fraction of DNA were as follows: first, 550 µL PEG6000 (300 g polyethylene glycol 6000, 93.6 g NaCl) was added, heated at 37 °C for 1 h or placed at 25 °C for 2 h, then centrifuged at 13,000 × g for 0.5 h, removed the supernatant, and cleaned with 0.5 mL 70% alcohol twice; in the last procedure of fractionation, the purified DNA was dissolved in 30 µL of nuclease-free water.

### Quantitative PCR (qPCR) analysis

QPCR was established on a Light Cycler 480 real-time PCR detection system (Roche480, USA) (Meng et al. 2020). The 515F/907R primer (Li et al. 2021a, b) was adopted for the bacterial 16S ribosomal(r) RNA gene, the CamoA-23F/616R (Meng et al. 2020) was adopted for the AOA *amoA* gene, and the amoA1F/2R (Meng et al. 2020) was adopted for AOB *amoA* gene. The six mixed primers comaA-244f_a-f/659r_a-f and comaB-244f_a-f/659r_a-f (Pjevac et al. 2017) were adopted for the comammox *Nitrospira* clade A (ComA) and comammox *Nitrospira* clade B (ComB) *amoA* genes, respectively (Table S1). The qPCR mixtures were 20 µL, containing 10 µL 2 × GoTaq® qPCR Master Mix (Promega), 0.5 M of each primer, and 2 µL of tenfold-diluted DNA template, and the part less than 20 µL was filled with nuclease-free water. The absolute quantitative PCR amplification conditions were as follows: The universal 16S rRNA gene was 95 °C for 3 min, followed by 35 cycles of 95 °C for 45 s, 56 °C for 45 s, and then 72 °C elongation for 5 min. The AOA were 95 °C for 2 min, followed by 40 cycles of 95 °C for 20 s, 55 °C for 20 s, and then 80 °C elongation for 30 s. The AOB were 95 °C for 2 min, followed by 40 cycles of 95 °C for 20 s, 57 °C for 30 s, and then 72 °C elongation for 30 s. ComA and ComB were 95 °C for 10 min, followed by 40 cycles of 94 °C for 30 s, 52 °C for 45 s, and then 72 °C elongation for 1 min. For qPCR, nuclease-free water was used as a negative control, the standard curve was established using a tenfold serial dilution of standard plasmid DNA, the amplification efficiencies were 93% to 99%, and the *R*^2^ was higher than 0.99.

### MiSeq sequencing and bioinformation

The microbial communities were paired-end amplicon sequenced, except for the *amoA* gene of AOA which used the forward-sequence because of it being lower than 635 bp; others used the data of paired-end amplicon sequence. The Illumina MiSeq (PE300) platform (Illumina, San Diego, USA) was run by Guangdong Magigene Biotechnology Co., Ltd. (Guangdong, China). In order to denoise (error-correcting) Illumina amplicon reads into unique sequences (zero-radius operational taxonomic units; Zotus), the UNOISE3 algorithm in USEARCH, which was updated from the UNOISE2 algorithm (Edgar 2016), was used, and the sequences are taxonomically classified. Sequence reads are archived in the NCBI Sequence Read Archive, with the Bioproject number PRJNA880760.

### Data analysis

The data of the nitrifying populations’ abundance was transformed to Log10 (base 10). In order to assess the differences, a one-way ANOVA (analysis of variance) was implemented. Graphs and histograms were drawn using the Origin 2017 software (Origin Lab Inc., USA). Phylogenetic analysis used the MEGA X version (Kumar et al. 2018). Bootstraps were based on 1000 replicated trees.

## Results

### Nitrification activity

**Fig. 1:**
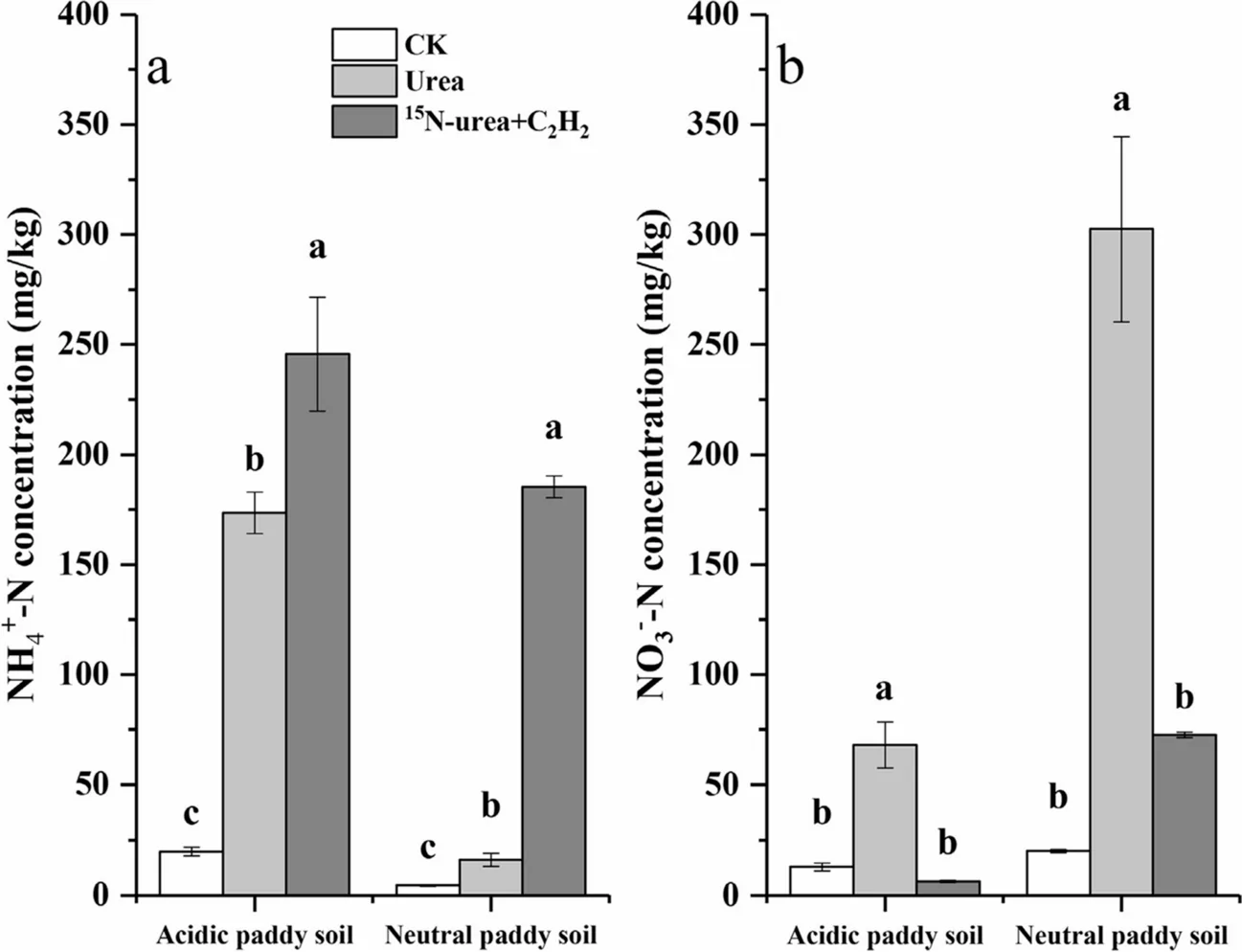
NH_4_^+^-N (**a**) and soil NO_3_^−^-N (**b**) concentrations following the microcosm incubation of the acidic and neutral paddy soils. Error bars indicate standard errors of triplicate samples. The urea treatment represents both ^14^N-urea and ^15^N-urea treatments as there was no difference between the two treatments

Soil nitrification was evaluated by the alterations in NH_4_^+^-N and NO_3_^−^-N contents (Fig. 1). The net nitrification rates for both soils were 2.10 and 5.24 mg kg^−1^ DWS day^−1^, respectively (Table 2). Urea fertilization caused a huge accumulation of NH_4_^+^-N in both soils (Fig. 1a). C_2_H_2_ additions significantly increased NH_4_^+^-N concentrations (*P* < 0.001) compared with the CK (Fig. 1a), Additionally, treated with urea alone was higher than the treated with urea and C_2_H_2_ at the significance level of 5% (Fig. 1a). Urea fertilization resulted in significant accumulation of NO_3_^−^-N in both soils (*P* < 0.001) with urea treatment (Fig. 1b). In both soils with added C_2_H_2_ inhibitor, NO_3_^−^-N concentrations were lower than those under urea alone at the significance level of 5% (Fig. 1b). The NH_4_^+^-N accumulation and NO_3_^−^-N production under C_2_H_2_ treatment indicated that autotrophic ammonia oxidation dominated in the both soils.

**Table 2:** The abundance and estimated cell activities of AOA and AOB in SIP microcosms

Soil*	DNA	Incubation time, day	Ratio of *amoA* genes			Copy numbers of *amoA* gene,			Net nitrification rate,
			in “HF” DNA to total DNA, %†			10^7^ g^−1^ dry soil			mg kg^−1^ dry soil day^−1^¶
			AOB	AOA	comammox *Nitrospira* clade A	AOB	AOA	comammox *Nitrospira* clade A	
Acidic paddy soil	Total DNA	0 days				0.11 ± 0.07	7.84 ± 0.87	6.39 ± 2.38	2.10 ± 0.46
		28-day urea				3.54 ± 2.74	24.1 ± 7.9	9.63 ± 8.99	
	^15^N-DNA	28-day urea	90.6 ± 1.0	87.1 ± 4.2		3.15 ± 2.44‡	21.3 ± 7.9‡		
Neutral paddy soil28-day urea	Total DNA	0 days				4.45 ± 1.47	29.6 ± 11.5	4.01 ± 0.53	5.24 ± 0.56
		28-day urea				14.4 ± 0.8	8.22 ± 1.35	3.52 ± 0.14	
	^15^N-DNA	28-day urea	93.0 ± 3.0		92.7 ± 2.8	13.3 ± 0.4‡		3.26 ± 0.14‡	

^*^Labeled microcosms with urea amendment in the absence of acetylene

^†^Ratio of gene copy numbers in the “heavy” DNA fraction (HF) (Fig. 3) to the sum of gene copies in all 16 DNA gradient fractions

^‡^Canonical ammonia oxidizers and comammox *Nitrospira* ^15^N-labeled *amoA* copies were calculated by multiplying the ratio of gene copy numbers in the “heavy” DNA fraction by the gene copy numbers of *amoA* in the total DNA at day 28 from each soil

^¶^Net nitrification rate in the ^15^N-labeled microcosms with urea amendment after incubation for 28 days

### Changes in the abundance

**Fig. 2:**
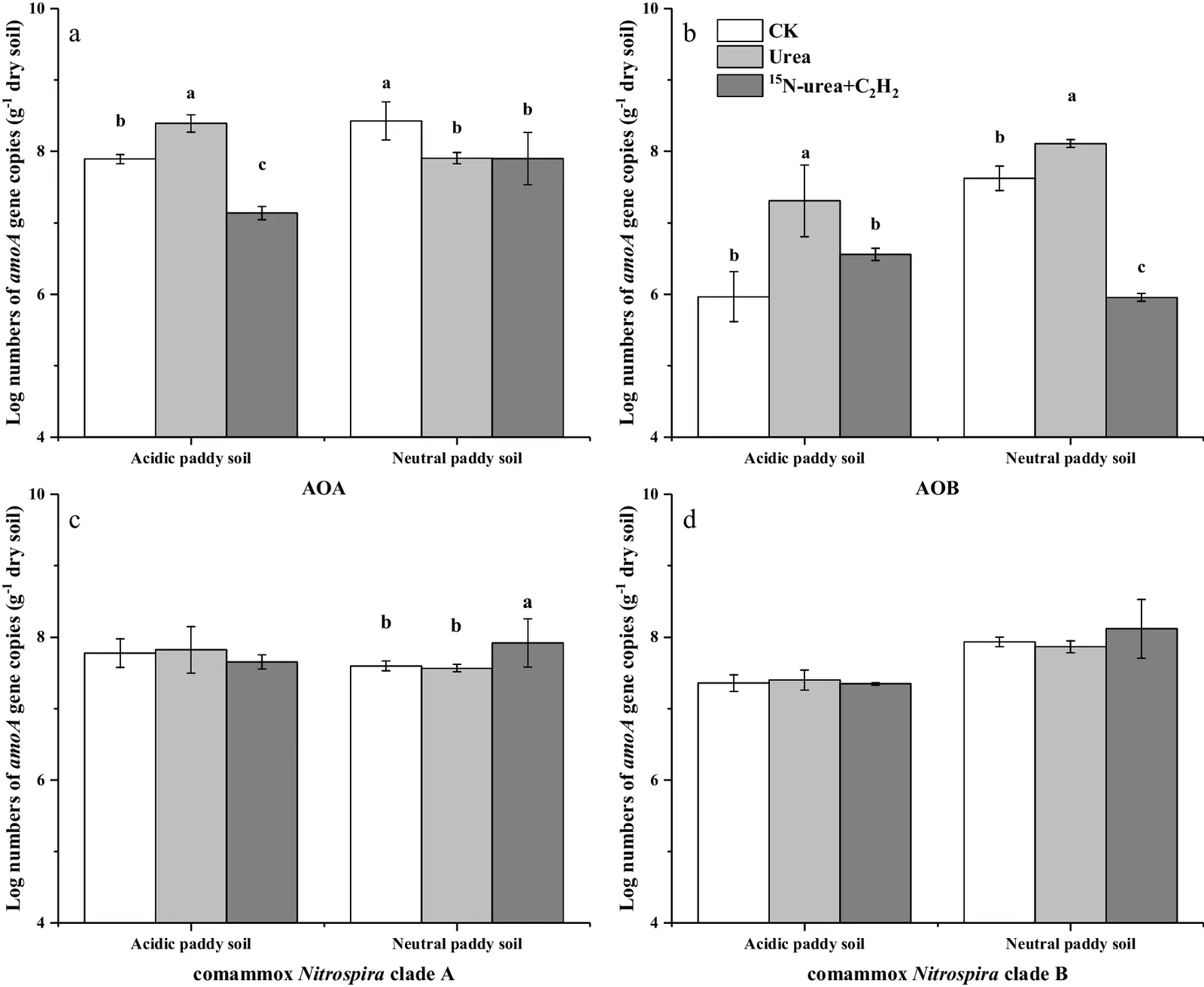
Changes in the *amoA* gene abundance (Log10) of AOA, AOB, and comammox *Nitrospira* clades A and B during the microcosm incubation of the acidic and neutral paddy soils. Error bars indicate standard errors of triplicate samples

qPCR of the *amoA* genes was performed to count the population abundance of AOA, AOB, ComA, and ComB (Fig. 2). The percentage of comammox *Nitrospira* in the nitrifying population (mean value: 44.3%) was lower than that of the canonical ammonia oxidizers, except for the treatment with C_2_H_2_ inhibitor in both soils (Fig. 2). On the contrary, the percentage of ComA and ComB in the nitrifying population in the C_2_H_2_ treatment (the highest value: 83.5%) was higher than that of AOA and AOB, due to the activities of the latter being significantly inhibited (Fig. 2).

Compared with the CK, the utilization of urea increased the AOA abundance in the acidic soil (*P* < 0.05). However, the AOA abundance in the neutral soil was significantly decreased (*P* < 0.05) (Fig. 2a). The existence of C_2_H_2_ inhibited the abundance in both soils, compared with the CK (Fig. 2a). Compared with the CK, the AOB abundance was enhanced by adding urea in both soils (Fig. 2b). Furthermore, the existence of C_2_H_2_ inhibited the abundance of AOB (Fig. 2b). Comammox *Nitrospira* abundances in the neutral soil were not significantly impacted by urea addition (Fig. 2c and d). However, C_2_H_2_ caused a significant increase in the ComA abundance (*P* < 0.001), possibly due to the activities of canonical ammonia oxidizers being significantly inhibited, while the ComB was unaffected (Fig. 2c and d).

### ^15^N-labeling ofactive ammonia oxidizers

**Fig. 3:**
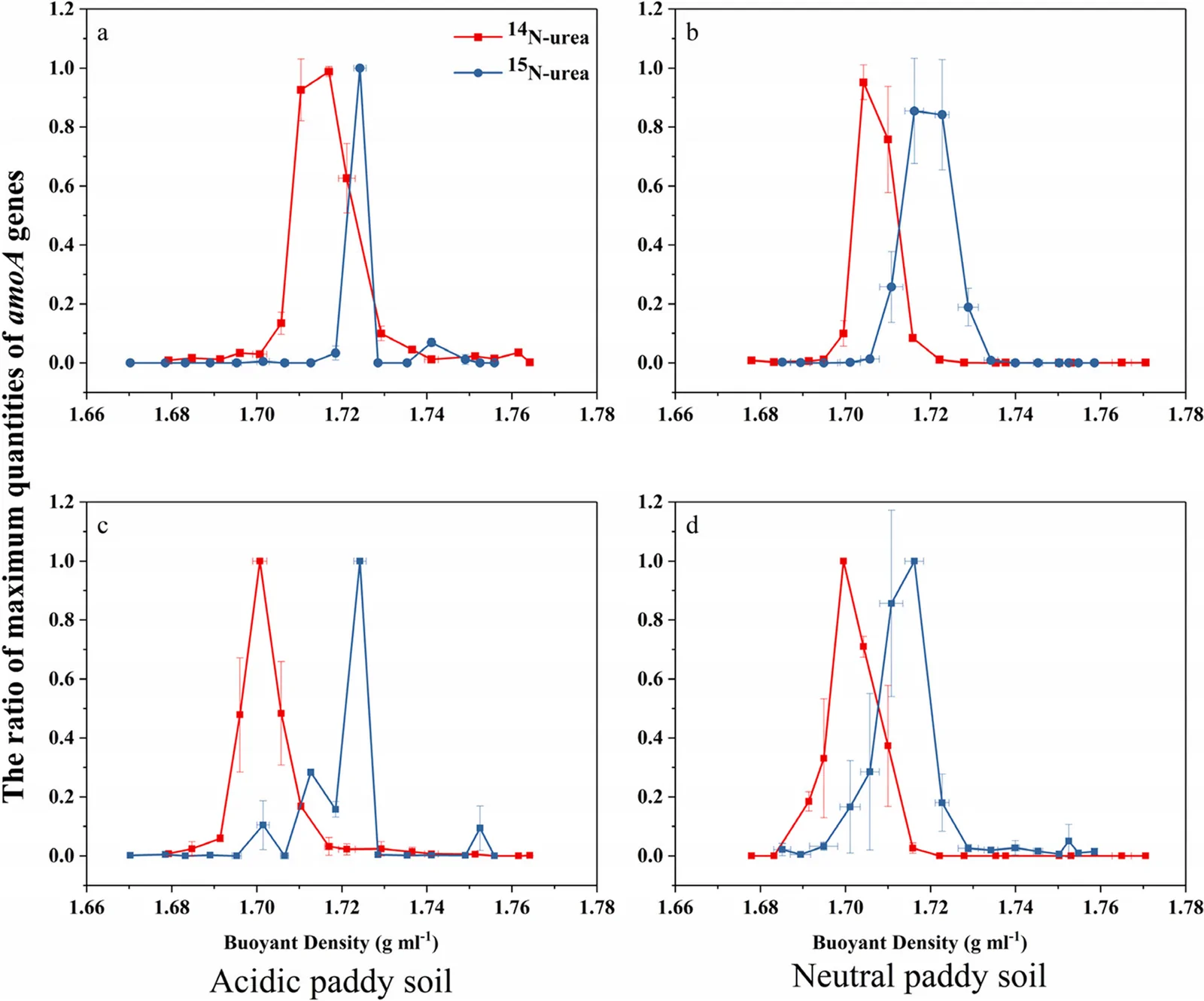
Quantitative PCR of gene copy numbers in the acidic soil of AOB (**a**) and AOA (**c**) and in the neutral soil of AOB (**b**) and comammox *Nitrospira* clade A (**d**) across the entire buoyant density gradient of the fractions from soil incubated with ^14/15^N-labeled urea at 28 days of incubation. The normalized data are the ratios of the gene copy number in each DNA gradient fraction to the maximum quantities from each treatment. Error bars show the standard errors of the triplicate microcosms

In order to distinguish the ^15^N-labeled DNA from the ^14^N-DNA, the activity of nitrifying populations was assessed by the quantification of *amoA* genes, which was isolated by density gradient centrifugation (Fig. 3). For the acidic soil, the peaks of AOA and AOB appeared in the ^15^N-labeled DNA ‘heavy fractions (HF)’ (fractions 7–9 and 6–7, respectively) from the ^15^N-urea microcosms when compared to those from the ^14^N-urea microcosms (Fig. 3a and c). For the neutral soil, the peaks of AOB and ComA also occurred in the ^15^N-labeled DNA “HF” (fractions 8–10 and 9–11, respectively) from the ^15^N-urea microcosms when compared to those from ^14^N-urea microcosms (Fig. 3b and. d). Although the active ^15^N-AOA appeared in DNA fractions 7–9, whereas the active ^15^N-AOB appeared in DNA fractions 6–7, the peak in HF for both occurred at a buoyant density (BD) of 1.7243 g mL^−1^ (Fig. 3a and c). Similarly, the active ^15^N-AOB appeared in DNA fractions 8–10, whereas active ^15^N-ComA appeared in DNA fractions 9–11; not only AOB but also ComA peaked at a BD of 1.7162 g mL^−1^ (Fig. 3b and d).

In the acidic soil, the percentage of gene copy number in the AOB and AOA HF to the sum in all 16 DNA gradient fractions was 90.6% and 87.1%, respectively (Table 2). In the neutral soil, the percentage of gene copy number in the AOB and AOA “heavy” DNA fractions to the sum in all sixteen DNA gradient fractions was 93.0% and 92.7%, respectively (Table 2). In the acidic soil, ^15^N-labeled AOB and AOA abundances were 3.15 × 10^7^ and 2.13 × 10^8^ copies g^−1^ DWS, respectively (Table 2). In the neutral soil, ^15^N-labeled AOB and ComA abundances were 1.33 × 10^8^ and 3.26 × 10^7^ copies g^−1^ DWS, respectively (Table 2).

### Population community of active ammonia oxidizers

**Fig. 4:**
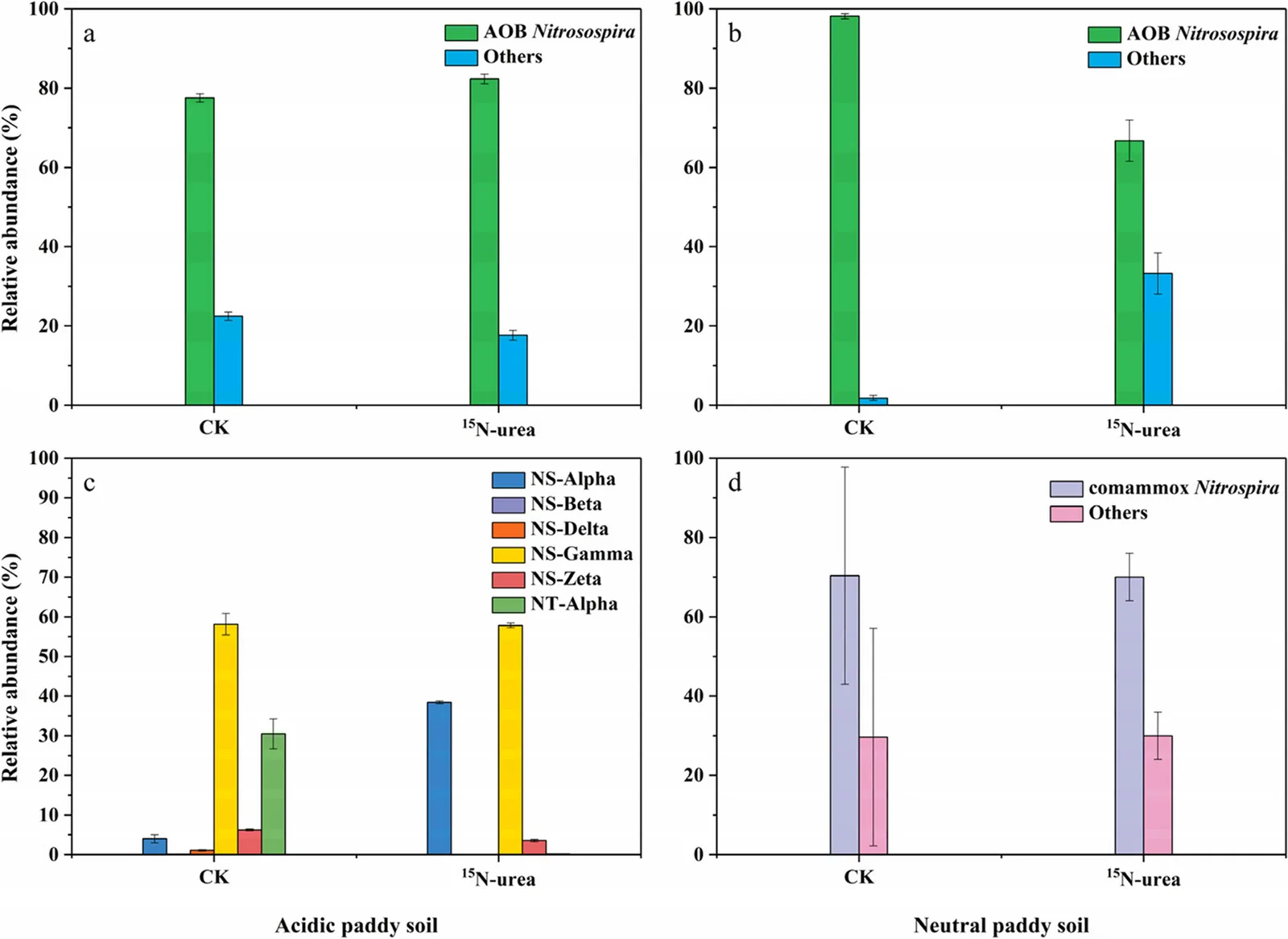
The population abundance of AOB (**a** and **b**), AOA (**c**), and comammox *Nitrospira* clade A (**d**) in the acidic and neutral rice soils. Zotus were clustered at 100% identity

All AOB fell within the *Nitrosospira* genus of the class β-Proteobacteria (Fig. 4a and. b). All AOA fell within the order of *Nitrososphaerales* and *Ca.* Nitrosotaleales (Fig. 4c). The AOA consisted of clades NS-Alpha, NS-Beta, NS-Delta, NS-Zeta, NS-Gamma, and NT-Alpha in the acidic paddy soil; the clades NS-Alpha (38.4%), NS-Gamma (57.8%), NS-Zeta (3.6%), NT-Alpha (0.2%), NS-Beta (0.04%), and NS-Delta (0.01%) exhibited autotrophic growth during urea microcosm incubation (Fig. 4c). Furthermore, NS-Alpha and NS-Gamma were the main active AOA (Fig. 4c), as suggested by the ^15^N-DNA-SIP. All comammox *Nitrospira* belonged to the *Nitrospira* genus (Fig. 4d).

**Fig. 5:**
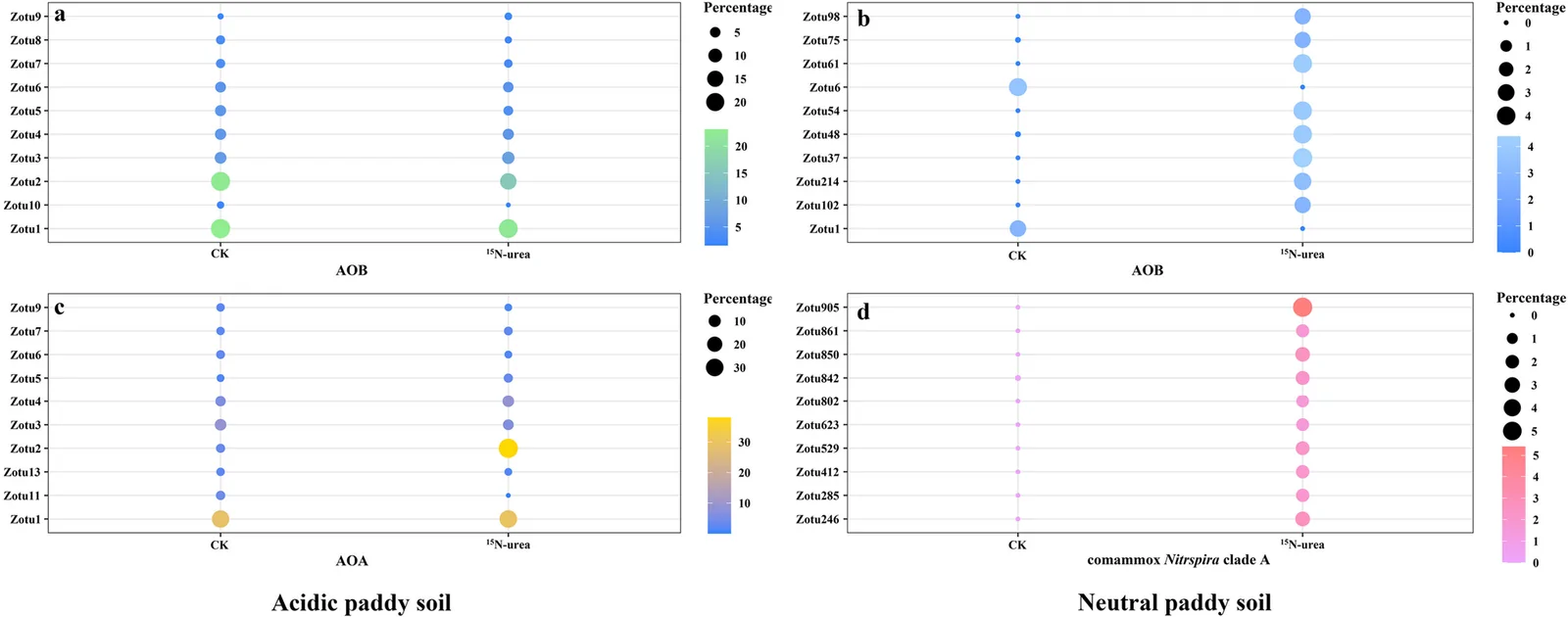
Ammonia oxidizing communities represented by the most abundant genera (top 10) in the heavy DNA fractions from the ^15^N-urea treatment and in the light DNA fractions from the CK in acidic and neutral paddy soils

Compared with the CK, the dominant Zotus in the AOB-active community demonstrated high consistency and significant differences in both soils (*P* < 0.05) (Fig. 5a and. b). The dominant Zotus in the active community of AOA was basically consistent with that in the control, except that Zotu2 showed dominance in the acidic soil (Fig. 5c). The dominant Zotus in the active community of ComA in the neutral soil was extremely different from that in the control (Fig. 5d). Meanwhile, we compared the difference of active AOB in both soils, that is, the nitrification activities in both soils were dominated by different Zotus (Table S2). This means that the AOB in both soils were different, even though they belonged to the same genus level.

**Fig. 6:**
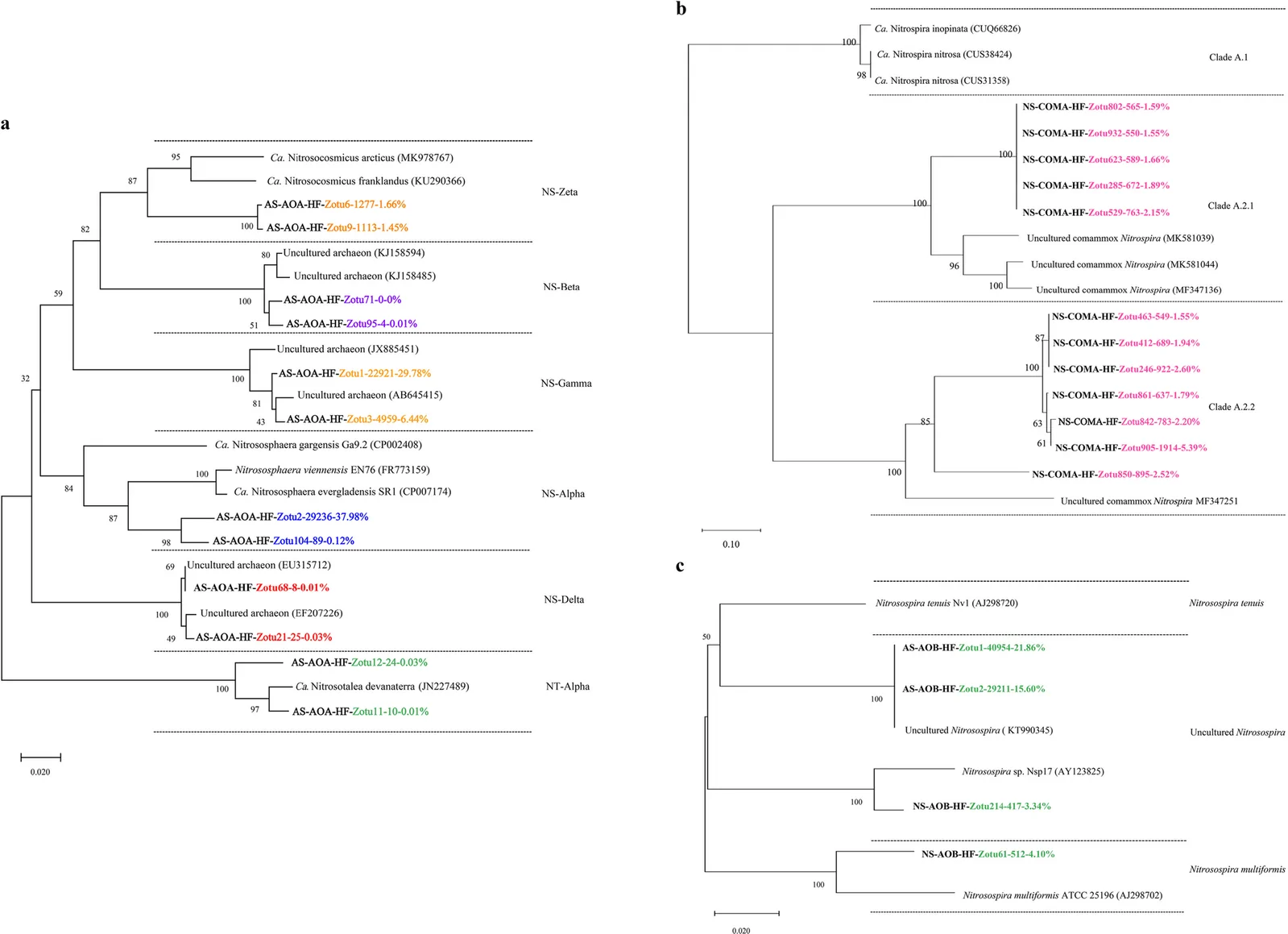
Phylogenetic analysis of AOA (**a**), comammox *Nitrospira* clade A (**b**), and AOB (**c**) *amoA* genes in ^15^N-labeled DNA from the ^15^N-urea-treated microcosms after an incubation period of 28 days. AS and NS represent acidic and neutral paddy soils, respectively. The designation “AS-AOA-HF–Zotu1-22,921–29.78%” indicates that Zotu1 contains 22,921 reads with 99% sequence similarity in the acidic paddy soil with the ^15^N-urea treatment. AS and NS mean acidic paddy soil and neutral paddy soil, respectively. Numbers with red, orange, yellow, green, blue, and purple colors represent sequences from different clades. NS and NT in notes mean Nitrososphaerales and *Ca.* Nitrosotaleales, respectively. The phylogeny of AOA, comammox *Nitrospira*, and AOB was generated using MEGA X with NJ-tree. Bootstraps are based on 1000 replicated trees. Bootstrap values are indicated at the branch nodes

The AOA *amoA* phylogeny includes four basic lineages, which are consistent with the taxonomic orders of class *Nitrososphaeria*, including *Nitrososphaerales* (NS) and *Ca.* Nitrosotaleales (NT) (Fig. 4). The active AOA mostly belonged to the NS (Fig. 6a). The *Nitrosocosmicus franklandus* and *Nitrososphaera viennensis* clusters (NS-Alpha) and some uncultivated archaea (NS-Gamma) dominated the active AOA in the acidic paddy soils, accounting for up to 38.10% and 36.22%, respectively (Fig. 6a). *Ca. Nitrosocosmicus arcticus*, *Ca. Nitrosocosmicus franklandus* cluster (NS-Zeta), and *Ca. Nitrosotalea devanaterra* (NT-Alpha) had some activity, though they only account for a small proportion. Most ComA *amoA* gene sequences affiliated with *Nitrospira* II were observed in the neutral paddy soil microcosms. Regarding the active comammox *Nitrospira* communities, most of the comammox *Nitrospira* labeled with ^15^N were classified into comammox *Nitrospira* clades A2.1 and A2.2 (Fig. 6b). The active AOB communities were controlled by the *Nitrosospira* genus of β-proteobacteria and members within the *Nitrosospira* genus lineage accounted for 40.8% and 7.4% of the ^15^N-labeled AOB *amoA* genes in the both soils, respectively (Fig. 6c). Moreover, the *Nitrosospira multiformis* and *Nitrosospira* sp. Nsp17 appeared in the neutral soil, accounting for 3.3% and 4.1% of the total AOB, respectively (Fig. 6c). Numerous ^15^N-labeled AOB *amoA* gene sequences were joined to uncultured *Nitrosospira* in the acidic soil (Fig. 6c).

## Discussion

Our results showed that active ammonia-oxidizing microorganisms changed significantly in the acidic and neutral soils under an intensive fertilization regime. Furthermore, since comammox *Nitrospira* were found, the growth and activity of each guild in ammonia oxidation under intensive fertilization regimes need to be re-evaluated. In this work, we utilized the ^15^N-DNA-SIP technique to uncover the activity of nitrifying populations.

An unsurprising finding was that AOA were higher than AOB in the acidic soil (Fig. 2a and b), especially after intensive fertilization. AOA were increased after urea application in the acidic soil at 5% significance level, whereas AOA were decreased after urea application in the neutral soil (Fig. 2a). Unsurprisingly, the AOA genome incorporated ^15^N during the incubation period in the acidic soil (Fig. 3a). Furthermore, the degree of AOA and AOB labeling suggested that AOA contributed significantly to the acidic soil (Fig. 3c), just as it was widely proposed that AOA play a significant role in autotrophic nitrification in acidic soils (Zhang et al. 2010; Yao et al. 2011; Li et al. 2018; Liu et al. 2021). A study based on soil pH gradient demonstrated AOA abundance and transcriptional activity, but the abundance and activity did not enhance with the reduction of pH (Nicol et al. 2008), and other results have elucidated that the AOA abundance was richer than that of AOB in acidic soils (Huang et al. 2012; Wu et al. 2017; Wang et al. 2019b). Nevertheless, the activity and growth of AOB in acid environments have been confirmed by the enrichment of AOB and the assimilation of ^13^CO_2_ (Huang et al. 2018; Picone et al. 2021; Bai et al. 2022). Indeed, under specific acidic conditions, AOB showed higher *amoA* gene abundance and transcriptional activity than AOA (Hayatsu et al. 2017), suggesting that pH is not a determining index in soil AOA and AOB niche under all conditions. A possible explanation for the continued activity of AOB in our acidic soil is that after urea application, urea hydrolysis can raise soil pH (Mehmood et al. 2018; Zhang et al. 2019), thereby creating an environment conducive to AOB growth, although subsequent nitrification can cause the pH to decrease again (Mehmood et al. 2018).

Another possible explanation for the activity of AOB is that the different ammonia oxidation pathways of AOB and AOA can affect the utilization of ammonium (Könneke et al. 2014; Kozlowski et al. 2016), accordingly influencing their abundance. The maximum activity (*V*_MAX_) and half-saturation constant (*K*_m_) of AOB were higher than that of AOA (Chen et al. 2018; Ouyang et al. 2018), which may lead to the rapid growth of AOB at high ammonium concentrations. In addition, the properties of the Rh-ammonia transporter may also be responsible for AOB abundance (Palomo et al. 2018), since ammonia levels in this study are millimolar at which AOB have a higher affinity and absorption capacity than AOA (Weidinger et al. 2007). The activity of AOB increases with the availability of ammonium (Ouyang et al. 2017), leading to a result in greater nitrification of AOB under conditions of high or saturated ammonia (Lin et al. 2021). For instance, AOA are more plentiful than AOB with lower ammonia contents in the open ocean (Wuchter et al. 2006; Agogué et al. 2008; Berg et al. 2015), whereas AOB response to ammonium was extremely significant in soil with higher ammonia concentrations (Verhamme et al. 2011; Palomo et al. 2018; French et al. 2021). This suggests that AOA were provided with much higher substrate affinity than AOB or comammox *Nitrospira* (Jung et al. 2021), that AOA are more plentiful in environments where ammonium is less available, and that AOB are more plentiful as the availability of ammonium increases (Herrmann et al. 2009; Auguet and Casamayor 2013; Jung et al. 2014). Hence, this research develops the understanding of the pH adaptability of AOB in paddy soil, particularly when ammonium concentrations are high.

ComA was not affected by fertilization, and apparently gave no labeling of ^15^N, while C_2_H_2_ had no effect (Fig. 2c), suggesting that comammox *Nitrospira* may be dormant. The same results also appeared in a ^13^CO_2_-labeled acidic soil (Liu et al. 2021), while the ComA abundance was not unaffected by urea (Liu et al. 2021; Feng et al. 2022). However, in another experiment with acidic forest soil, ComA incorporated ^13^CO_2_ (Li et al. 2019, 2020). Recent studies have revealed that the ComA is highly similar to the Rh-type transporters which were found in almost all β-AOB (Palomo et al. 2018; Gao et al. 2022). The Rh-type ammonia transporter can exhibit affinity for ammonium with high absorption capacity within the range of millimole (Palomo et al. 2018), which may favor the autotrophic growth of ComA at high ammonium concentrations. Therefore, ComA can undertake NH_4_^+^ to NO_3_^−^ in acidic soil under specific circumstances. The activity of the neutral soil was verified by ^15^N. ^15^N was labeled, indicating that ComA participated in ammonia oxidation in the neutral soil (Fig. 3d). However, the low proportion of it labeled with ^15^N suggested that they played a lesser influence over autotrophic nitrification than canonical ammonia oxidizers (Table 2). Under high ammonium availability, the ability of ComA to absorb urea may lead to a change in competitiveness, which may be weaker compared to AOA and/or AOB (Liu et al. 2021), also having a *K*_m_ (NH_3_) in comammox *Nitrospira* of only 63 nM (Kits et al. 2017). In the neutral soil, the ComA was observed with ^15^N-urea labeling (Gao et al. 2022), and this study supports evidence from previous observations that it grew autotrophically (Li et al. 2019; Zhao et al. 2020; Liu et al. 2021). All current comammox *Nitrospira* strains were isolated from aquatic systems, and comammox *Nitrospira* grows preferentially in neutral to slightly alkaline conditions (Daims et al. 2015; van Kessel et al. 2015; Li et al. 2019). A recent study provides direct and reliable evidence for nitrification that is partially due to neutral or basophilic ComA in soil. Since the average C/N ratio in the genome is about 2.1:1 (Cadisch et al. 2005) and the BD of the ^15^N-labeled fraction is only increased by about 0.02 g mL^−1^ (Cupples et al. 2007), we excluded the growth of ComB using ^15^N-urea in both soils. After nitrogen application, the autotrophic growth of ComB could not be detected (Fig. 2d), which might be the result that ComB only grows in paddy soils without nitrogen input (Wang et al. 2019a). Additionally, as weekly urea addition was unlikely to be the limiting factor for ComB growth, the absence of ComB growth suggests a potential inhibition, perhaps due to higher nitrogen input (Wang et al. 2019a; Jiang et al. 2020).

The AOB genome was more labeled with ^15^N than ComA in the neutral soil (Fig. 3b and. d). Additionally, AOB was significantly increased by applying urea, while urea application had no effect on ComA (Fig. 2b and. c). This indicated that AOB had a stronger contribution than ComA in the neutral soil. Although this method is a preliminary assessment of the relative contribution of ComA (Li et al. 2019), this finding still contributes to our understanding of the relative contribution of the nitrifying population.

AOA contain diverse organisms, including the NC, NS, NT, and NP. However, these organisms are extremely underrepresented in currently cultivated strains and genomes, compared to the total AOA yield (Alves et al. 2018). All AOA belonged to the class *Nitrososphaeria* (Fig. 4c); this also accords with earlier observations, which showed that *Thaumarchaeota* are the predominating lineage in acid soils (Subrahmanyam et al. 2014; Wu and Conrad 2014; Dai et al. 2018). NS-Alpha, NS-Beta, NS-Gamma, NS-Delta, NS-Zeta, and NT-Alpha exhibited autotrophic growth with different yields during urea miniature culture in the acidic soil (Fig. 4c). Only a few lineages of AOA occurred in acidic soils with pH below 7.0, including lineage NT and a few NS clades, particularly NS-Alpha and NS-Gamma (Alves et al. 2018), which was in agreement with our result that the AOA activity was dominated by NS-Alpha and NS-Gamma, as showed by the ^15^N-DNA-SIP (Fig. 4c). These results suggested that most AOA in the paddy soil showing autotrophic growth, which was identified using the *amoA* gene in the DNA-SIP, always originated from the AOA closely similar to the strains cultivated (Zhao et al. 2015; Alves et al. 2018; Pan et al. 2019). Moreover, Zotu2 dominated in the control treatment (Fig. 5c), Zotu1 and Zotu2 performed a dominant role in the ^15^N-urea treatment (Figs. 5c and. 6a), which came from NS-Gamma and NS-Alpha (Fig. 6a), respectively, indicating their dominant contribution to the growth of the AOA community. One interesting finding was that Zotu2 was consistently present and dominant in both control and ^15^N-urea treatments, suggesting that the clade of NS-Alpha could adapt to environmental changes and may maintain AOA diversity and activity in soils that are disturbed, for example, during intensive fertilization.

In either acidic or neutral soils, the AOB was from the genus *Nitrosospira*, and no traces of *Nitrosomonas* and *Nitrosococcus* were found (Fig. 4a and. b). Interestingly, the dominant Zotus differed in both soils (Fig. 5a and. b, Table S2). The phylogenetic analysis further reflected that the dominant Zotus in the acidic and neutral soils belonged to different AOB clades, respectively (Fig. 6c). These findings suggested that AOB-active taxa in the acidic soils differed from those in the neutral soils at the OTU level. It can help us to understand the survival patterns and population dynamics of AOB in acidic and neutral soils and provide strong support for explaining the dominant role of AOB under high nitrogen input.

ComA dominated ammonia oxidation in the neutral paddy soil but may play a lesser role in nitrification than AOB. Additionally, the dominant Zotus in the ^15^N-urea treatment was different from that in the control, and the proportions of each Zotus were also similar (Fig. 5d), indicating the option of these Zotus for high nitrogen input. Based on phylogenetic analysis, ComA was previously divided into four subclades, clades A.1, A.2.1, A.2.2, and A.3 (Li et al. 2021a, b). Two comammox *Nitrospira* clusters were found in this study, ComA.2.1 and ComA.2.2 (Fig. 6b), commonly obtained from terrestrial ecosystems (Sun et al. 2021a, b; Yuan et al. 2021). Comammox *Nitrospira* community was predominated by ComA.2.2, which was different from other studies where ComA.2.1 dominated (Lin et al. 2022; He et al. 2022), probably because ComA.2.1 was biased towards relatively high pH (Lin et al. 2022; He et al. 2022). However, due to the lack of cultures, the survival mechanisms of ComA.2.1 and A.2.2 in agricultural ecosystems are still unclear and should be considered in future studies.

In this study, ^15^N-urea was used as a tracer to reveal the active taxa within both paddy soils. Despite the current high level of fertilization in the field, with the high demand for agricultural production, continued intensive fertilization may be expected to be applied to the soil in pursuit of high yields. Therefore, this experiment provides a prediction for future changes in the microbial populations following fertilizer application. It may be due to the continuous nitrification during the extraction process, the measured ammonium concentration may be lower than the original level of the soil, or the strong adsorption of the soil caused the ammonium concentration to be too low (Wang and Alva 2000; He et al. 2021; He et al. 2021). In addition, the combined effect of organic nitrogen mineralization in the soil and the oxidation of ammonium can also cause the measured ammonium concentration to be lower than the actual level (Wang et al. 2018). Furthermore, we elucidate differences in active taxa in both paddy soils at the OTU level. However, the sequencing primers and the specificity of the comammox *Nitrospira* gene under aerobic conditions need to be further developed and improved to make the characterization of the ammonia-oxidizing microbial community more direct. Meanwhile, the specific nitrogen cycle processes for comammox *Nitrospira*, AOA, and AOB coupling are still unclear, and more experiments are needed to further focus on functional genes and microbial metabolic processes related to the nitrogen cycle.

The study showed that urea significantly enhanced the abundance of canonical ammonia oxidizers in both soils, and the existence of C_2_H_2_ almost completely inhibited nitrification and the activities of these groups in both soils. ^15^N-DNA-SIP plays a useful role in distinguishing nitrifying populations and revealing their niche differentiation. ComA incorporated ^15^N into the genome, showing that it was active in the neutral soil. Furthermore, the low proportion of it labeled with ^15^N suggests that it plays a lesser role in autotrophic nitrification than canonical ammonia oxidizers. The growth of canonical ammonia oxidizers and ComA was detected, and it illuminated that canonical ammonia oxidizers dominated ammonia oxidation in the acidic soil, while AOB and ComA dominated in the neutral soil. In particular, although AOB grew autotrophically in both soils, phylogenetic analysis showed that AOB-active taxa differed from those at the OTU level. These findings develop our understanding of the nitrogen cycle and indicate that the ecological importance of nitrifying populations needs to be reassessed in environments impacted by nitrogen input.

## Supplementary Information

Below is the link to the electronic supplementary material.

Supplementary file1 (XLSX 18 KB)

## Data Availability

All datasets generated for this study are included in the article/supplementary material.
